# A Cross-Sectional Study on the Association between 24-h Urine Osmolality and Weight Status in Older Adults

**DOI:** 10.3390/nu9111272

**Published:** 2017-11-22

**Authors:** Patrícia Padrão, Ana S. Sousa, Rita S. Guerra, Luísa Álvares, Alejandro Santos, Nuno Borges, Cláudia Afonso, Teresa. F. Amaral, Pedro Moreira

**Affiliations:** 1 Faculdade de Ciências da Nutrição e Alimentação da Universidade do Porto, 4200-465 Porto, Portugal; sofia.limas.sousa@gmail.com (A.S.S.); ritacsguerra@gmail.com (R.S.G.); luisa.m.alvares@gmail.com (L.Á.); alejandrosantos@fcna.up.pt (A.S.); nunoborges@fcna.up.pt (N.B.); claudiaafonso@fcna.up.pt (C.A.); tamaral@fcna.up.pt (T.F.A.); pedromoreira@fcna.up.pt (P.M.); 2 EPIUnit—Instituto de Saúde Pública, Universidade do Porto, 4050-600 Porto, Portugal; 3 Escola Superior de Saúde de Leiria, Instituto Politécnico de Leiria, 2411-901 Leiria, Portugal; 4 I3S-Instituto de Investigação e Inovação em Saúde, 4200-135 Porto, Portugal; 5 CINTESIS—Centre for Health Technology and Services Research, 4200-450 Porto, Portugal; 6 System Integration and Process Automation Unit (UISPA)-Institute of Mechanical Engineering (IDMEC), Faculdade de Engenharia, Universidade do Porto, 4200-465 Porto, Portugal; 7 Centro de Atividade Física, Saúde e Lazer, Universidade do Porto, 4200-450 Porto, Portugal

**Keywords:** hydration, urine osmolality, weight status, obesity, body mass index, elderly

## Abstract

Data on the association between hydration and body weight in the elderly are scarce. The objective of this work was to quantify the association between 24-h urine osmolality and weight status in the elderly. A cross-sectional study was conducted within the Nutrition UP 65 study. A quota sampling was implemented to achieve a nationally representative sample of Portuguese older adults (≥65 years) according to age, sex, education and region. From a sample size of 1500 participants, 1315 were eligible for the present analysis, 57.3% were women and 23.5% were aged ≥80 years. Participants were grouped using tertiles of 24-h urine osmolality by sex. World Health Organization cutoffs were used to classify participants according to weight status. Multinomial multivariable logistic regression models were conducted to evaluate the association of tertiles of osmolality with weight status, adjusting for confounders. Odds Ratios (OR) and respective 95% Confidence Intervals (95% CI) were calculated. Being in the 3rd urine osmolality tertile (highest) was associated with a higher risk of being obese in men, OR = 1.97, 95% CI = 1.06, 3.66. No such association was found in women. These results highlight the need for implementing studies in order to clarify the association between hydration and weight status in the elderly.

## 1. Introduction

Obesity is associated with diverse co-morbidities, including type 2 diabetes, hypertension, coronary artery disease, various types of cancer, cognitive dysfunction [1], gallbladder disease, osteoarthritis and asthma [2]. In the last few decades, obesity has been growing in importance as the leading risk for disability adjusted life years (DALYs) [3]. At the same time, a rapid rise in life-expectancy has been observed in most countries, increasing the health care challenges in the ageing population [2], despite the fact that prevalence of obesity is lower among older adults compared to younger ages [4].

Hypohydration, the state of being in negative water balance (water deficit) [5] is also a frequent condition among older people, being estimated to increase the odds towards higher morbidity and mortality in this age group [6]. It is a frequent etiology of hospitalization of the elderly, increasing the risk of infection [7]. However, a difficulty is that there is no universally accepted method for measuring hydration status, especially in large-sample studies [8]. This contributes to the scarcity of research on the topic [9,10] despite the clues about the importance of good hydration for the prevention of chronic diseases [9,10]. To assess acute hydration changes, plasma osmolality constitutes an appropriate marker while urinary markers appear to be the most adequate for detecting mild hypohydration in large populations [8]. Shirreffs argued that urine indices, and in particular urine osmolality, is the most promising marker available to assess hydration status, despite seeming to fail in identifying changes in hydration status during periods of rapid body fluid turnover [5]. There are notable differences in 24-h urine osmolality between healthy subjects mostly caused by age, renal solute excretion, sex and cultural context [11] and there is no consensual threshold for classifying the hydration status of individuals according to urine osmolality. Several cut-offs of urine osmolality have been proposed to classify subjects according to their hydration status, such as 500 mOsm/kg [12], 800 mOsm/kg [13] or 850 mOsm/kg [14], but only the concept of Free Water reserve proposed by Manz et al. [11] takes into account the loss of renal capacity with aging.

An association between a low usual fluid intake and some chronic diseases has been described, including urolithiasis, constipation, asthma, cardiovascular disease, diabetic hyperglycemia, and some cancers, even though it is unclear if there is any causal relation [7].

Data regarding the association between hydration status and obesity carried out in older adults are lacking. Notwithstanding this, some studies conducted in younger adults’ samples have shown important results. Findings from two recent published studies using the National Health and Nutrition Examination Survey (NHANES) cross-sectional survey 2009–2012 showed that inadequate hydration assessed with urinary biomarkers was associated with higher BMI among adults [13,15], and although Rosinger et al. included elderly, the results were not stratified by age [15]. Chang et al. highlighted the need to perform additional research on the topic and suggested an inadequate water intake in obese adults, namely through a lower intake of foods rich in water such as fruit and vegetables [13], despite no association of total water intake with all-cause mortality [16].

It is expected that older adults are exposed to a higher risk of hypohydration when compared to younger adults due to physiologic changes including the decrease in perception of thirst and decrease in renal function (5–9). Therefore, the objective of this work was to quantify the association between 24-h urine osmolality and weight status in a representative sample of Portuguese older adults.

## 2. Participants and Methods

The study followed the reporting guidelines of the STROBE statement for cross-sectional studies.

### 2.1. Study Design and Sampling

A cross-sectional observational study was conducted in Portugal on a sample of 1500 older Portuguese, ≥65 years old. The study sample was composed of 95% of community-dwelling older adults and of 5% by individuals institutionalized in retirement homes. This proportionality of 5% was previously described for older Portuguese individuals institutionalized in retirement homes [2].

A nationally representative sample of Portuguese older adults was achieved, regarding sex, age, educational level and regional area (defined in the Nomenclature of Territorial Units for Statistical purposes—NUTS II).

Data from the most recent national census in 2011 showed that the number of Portuguese residents was 10,562,178 and that 2,010,064 (19%) were aged ≥65 years [2]. Then, a study sample of 1500 older adults equivalent to 0.075% of the Portuguese older population was defined. Considering the population structure in terms of sex, age and education level, the number of subjects in each strata of the regional area was ascertained (Please see Multimedia Appendix 1 of Nutrition UP 65 description) [17].

In each one of the seven regional areas (NUTSII), town councils with >250 inhabitants were identified and three or more were randomly selected. Potential community dwelling participants were contacted via home approach, telephone, or via institutions, such as town councils and parish centers. Individuals were eligible to participate in this study if they were Portuguese, aged ≥65 years and not presenting any condition that precluded the collection of venous blood samples or urine (e.g., dementia or urinary incontinence). They were considered to be Portuguese if they had only Portuguese nationality and if their current tax residence was in Portugal. Potential participants fulfilling these conditions were invited to participate and some volunteered. They were recruited until the number of subjects fulfilling the characteristics of the pre-defined sample was reached [17].

The interviewers provided information about the study purposes and the methodology when inviting the potential participants. Individuals presenting any condition that precluded the collection of urine, such as dementia or urinary incontinence, were excluded from the study.

Data were collected between December 2015 and July 2016.

### 2.2. Data Collection

A face-to-face interview using a structured questionnaire was conducted by eight previously trained registered nutritionists to collect information on sociodemographic characteristics, lifestyle, cognitive performance, physical activity, and nutritional status.

Socio-demographic data included information on sex, age, regional area, education, marital status, residence type and household income. The regional areas used are defined in NUTS II: Alentejo, Algarve, Azores, Lisbon Metropolitan Area, Center, Madeira and North [18]. Educational level was determined by the number of completed school years and the following categories were used: no formal education, 1–3, 4, 5–11 and ≥12 years of school. Marital status was categorized as single, married or in a common-law marriage, divorced or widowed. For analysis, participants were grouped into two categories: single, divorced or widowed, and married or in a common-law marriage. Residence type was defined as home or institution.

Household monthly income was summarized using the following cut-offs: <€500, €500–999 and ≥€1000. Among the included participants, 645 (48.9%) did not know or preferred not to declare their income and thus they were allocated in a separate category.

Lifestyle was assessed by information on physical activity, tobacco use and alcoholic beverage consumption. The short form of the International Physical Activity Questionnaire (IPAQ) [19], which concerns activities performed during the previous seven days, was used to assess physical activity. Data collected with IPAQ were converted to MET-minutes. Median values were calculated for walking, moderate-intensity activities, and vigorous-intensity activities using established formulas. Total physical activity Metabolic Equivalent of Task (MET), MET-min/week was defined as the sum of walking + moderate + vigorous MET-min/week scores [20]. MET-min scores are equivalent to kilocalories for a 60 kg person. Kilocalories were computed from MET-min/week scores [19] and individuals were classified as either presenting low physical activity levels, <383 kcal/week (men) and <270 kcal/week (women), or as presenting normal physical activity levels (≥383 kcal/week and ≥270 kcal/week, respectively for men and women) [21]. Alcoholic beverage consumption was assessed by the number of drinks per day: moderate alcoholic consumption was defined as 1 drink/day for women and as 1 or 2 drinks/day for men, whereas heavy consumption was defined as ≥2 drinks/day for women and as ≥3 drinks/day for men.

Cognitive performance was assessed by the Portuguese version of the Mini Mental State Examination [22]. Cognitive impairment was defined as follows: individuals with no education, ≤15 points; 1 to 11 years of years of school completed, ≤22 points; and >11 years of school completed, ≤27 points [22]. Each participant’s nutritional status was evaluated by the assessment of the anthropometric measurements body weight and standing height. Body mass index (BMI) was computed using the standard formula (body weight (kg)/standing height^2^ (m)). World Health Organization cutoffs were used to define underweight (<18.5 kg/m^2^), normal weight (18.5–24.9 kg/m^2^), overweight (25.0–29.9 kg/m^2^) and obesity (≥30.0 kg/m^2^). Undernutrition status was assessed by the Portuguese version of the Mini-Nutritional Assessment^®^-Short Form (MNA^®^-SF) [23,24]. Only two participants were classified as underweight and therefore underweight and normal weight participants were grouped in one category.

Anthropometric data were also collected by the interviewers. All the anthropometric measurements were collected following the International Standards for Anthropometric Assessment (ISAK) procedures [25]. Standing height was obtained with a calibrated stadiometer (Seca 213, SECA GmbH, Hamburg, Germany) with 0.1 cm resolution. For participants with visible kyphosis or when it was impossible to measure standing height due to participant’s paralysis or due to mobility or balance limitations, height was obtained indirectly from non-dominant hand length (in centimetres), measured with a calibrated paquimeter (Fervi C056, FERVI, 20 Vignola, Italy) with 0.1 cm resolution [26]. Body weight (in kilograms) was measured with a calibrated portable electronic scale (Seca 803, SECA GmbH, Hamburg, Germany) with 0.1 kg resolution, with the participants wearing light clothes. Anthropometric assessment (including weight) occurred after the completion of the questionnaires, and participants were not asked to empty the bladder before weighing. When it was not possible to weigh a patient, for the same reasons referring to the standing height measurement, body weight was estimated from mid-upper arm and calf circumferences [27]. Mid upper arm, waist and calf circumferences were measured with a metal tape measure from Lufkin with 0.1 cm resolution. Participants were classified according to the following waist circumference categories: no risk (women: <80 cm; men: <94 cm), high risk (women: ≥80 cm and ≤88 cm; men: ≥94 cm and ≤102 cm), and very high risk (women: >88 cm; men >102 cm) [28].

Given the possible association between medication and supplement use and hydration status, the number of medicines and supplements taken chronically was assessed by self-reporting and participants were categorized as follows: 0 or unknown use, 1–4 and ≥5 medicines and supplements/day. Specifically, the use of diuretics was also assessed.

The volume of urine in a 24-h period was collected for each participant. The interviewers gave the participants detailed oral and written instructions (a leaflet was distributed to all participants) on how to proceed for a valid collection and adequate storage of the volume of 24-h urine. Participants were taught to discard the first morning void and to collect all urine over the following 24-h, including the first void in the following morning, and to keep note of the time of the start and end of collection. A 24-h urine 3 L container was also provided. The instructions for the collection of the 24-h urine samples were given after the interviews. A certified laboratory, Labco Portugal, was responsible for urine sample collection and analyses. Analyses included the quantification of urine volume (mL), urinary creatinine (mg/day) and urine osmolality (mOsm/kg). Urinary creatinine was measured by the Jaffe method. A urine sample was considered inadequate if the creatinine level was <0.4 g/24-h for women and <0.6 g/24-h for men [29] or if the volume collected was <500 mL [30].

For analysis, urine osmolality was summarized using tertiles by sex, according to the cutoffs of sample distribution stratified by sex. For women, cutoffs were <360.1 mOsm/kg, ≥360.1 and <484.8 mOsm/kg, and ≥484.8 mOsm/kg; for men, the cutoffs were <433.2 mOsm/kg, ≥433.2 and <602.1, and ≥602.1 mOsm/kg.

Participants were grouped into two categories according to season of sample collection: Autumn/Winter and Spring/Summer.

### 2.3. Statistical Analysis

Categorical variables were reported as frequencies. According to the normality of variables distribution, evaluated through Kolmogorov-Smirnov test, BMI and 24-h urine volume were described as the median and interquartile range (IQR). Sex differences were compared with the Mann-Whitney test.

Participants were compared across tertiles of osmolality for several socio-demographic, lifestyle, clinical, anthropometric and nutritional characteristics using the Kruskal-Wallis test for continuous variables, and Pearson qui-square test for categorical variables.

Multinomial multivariable logistic regression models were conducted to evaluate the association of tertiles of osmolality with weight status, adjusting for possible confounders of this association. Odds Ratios (OR) and respective 95% Confidence Intervals (95% CI) were calculated.

The Kruskal-Wallis test was performed to compare urine osmolality medians across BMI and waist circumference categories.

Results were considered significant when *p* < 0.05. Statistical analyses were conducted using the Statistical Package for Social Sciences for Windows (version 23.0, 2012, IBM-SPSS, Inc., Chicago, IL, USA).

### 2.4. Sensitivity Analysis

We further tested the association between urine osmolality and weight status excluding participants with self-reported diabetes (*n* = 361) and those taking diuretics (*n* = 200).

### 2.5. Ethics

This research was conducted according to the guidelines established by the Declaration of Helsinki and the study protocol was approved by the Ethics Committee of the department of “Ciências Sociais e Saúde” (Social Sciences and Health) from the “Faculdade de Medicina da Universidade do Porto” (No. PCEDCSS—FMUP 15/2015) and by the Portuguese National Commission of Data Protection (No. 9427/2015). All participants, or two representatives by participant in case of cognitive impairment, were asked to read and sign a duplicated “Informed consent” form.

## 3. Results

Of the 1500 subjects recruited, 178 participants were excluded because urine samples were inadequate. For the other four participants, it was either not possible to measure or to estimate weight. Therefore, this study final sample is composed of 1318 participants, median (IQR) age equal to 73 (10) years, between ages 65 and 94 years, 57.3% women. Within this sample of older adults, height was estimated from hand length for 25 participants and weight was estimated from from mid-upper arm and calf circumferences for 12 participants.

For women, median (IQR) BMI was 29.5 (6.2) kg/m^2^ whereas for men these values were 28.4 (5.2) kg/m^2^ (*p* < 0.001). Almost half of the study participants were overweight, *n* = 598 (45.4%) and almost two fifths were obese, *n* = 519 (39.4%).

Characteristics of the participants across tertiles of osmolality and according to sex, respectively for women and men, are presented in Table 1 and Table 2.

Higher proportions of women from the North region, community-dwelling and with normal physical activity were found in the highest osmolality tertile (Table 1). In men, higher proportions of participants aged 65–79 years old, living in the community, married or in a common-law marriage and with no use or unknown use of diuretics were found in the highest osmolality tertile (Table 2).

For women, median (IQR) urine volume was 1550 (700) mL whereas for men these values were 1700 (750) mL (*p* < 0.001).

Median urine osmolality was observed to increase with BMI categories both in women (underweight/normal weight: 395 mOsm/kg; overweight: 406 mOsm/kg; and obesity: 435 mOsm/kg, *p* = 0.003) and men (underweight/normal weight: 453 mOsm/kg; overweight: 469 mOsm/kg; and obesity: 530 mOsm/kg, *p* = 0.026).

Regarding waist circumference, median urine osmolality was higher in women classified in the very high risk group (no risk: 389 mOsm/kg; high risk: 383 mOsm/kg; very high risk: 451 mOsm/kg, *p* = 0.008). No such association was found in men (no risk: 483 mOsm/kg; high risk: 473 mOsm/kg; very high risk: 505 mOsm/kg, *p* = 0.797).

Significant differences according to geographic region (categorical), residence type (categorical dichotomous) and physical activity (categorical dichotomous) were found between tertiles of osmolality in women, so these variables were used in the multivariable analysis. In men, significant differences between osmolality tertiles were found for age (categorical dichotomous), residence type (categorical dichotomous), marital status (categorical dichotomous) and use of diuretics (categorical dichotomous) and thus these were the variables included in the multivariable procedure. Being in the 2nd or in the 3rd (highest) osmolality tertiles was not associated with overweight or obesity in women. For men, being in the 2nd osmolality tertile was not associated with overweight or obesity; being in the 3rd tertile (highest) was not associated with overweight but was associated with a higher risk of being obese, OR = 1.97, 95% CI = 1.06, 3.66 (Table 3).

The results were consistent with the primary analyses when participants with diabetes and those taking diuretics were excluded.

## 4. Discussion

In this study conducted on a representative sample of older Portuguese, obesity was associated with higher urine osmolality among men. A recent study conducted within the National Health and Nutrition Examination Survey (NHANES) survey 2009–2012 showed, for the first time at the population level, a significant association between inadequate hydration and obesity among adults of both sexes [13]. This study was methodologically different from the present study, namely regarding the age group addressed, in which the NHANES study included adults under 65 years, and also the cut-off used to classify subjects according to hydration status (subjects with urine osmolality of 800 mOsm/kg or above were considered to be inadequately hydrated). Nevertheless and despite these important differences, both studies suggested an association between hydration and obesity, which should be taken into account in research and clinical contexts. Changed fluid distribution reflected in a higher volume of extracellular to intracellular fluid ratio has been described in obese when compared to normal weight subjects [31] though its implications in hydration status remain unclear.

Rosinger et al. recently studied the role of obesity in the relation between total water intake and urine osmolality in US adults aged ≥20 years as a whole [15], and concluded that obesity modifies the association between water intake and hydration status. The authors showed that obese adults may need to consume more water than underweight/normal weight in order to exhibit similar hydration gains which may not occur in sufficient quantity to cover the increased needs [15]. Although we did not assess dietary intake in the present survey, a lower consumption of vegetable soup and fruit has been described to be associated with obesity in a representative sample of Portuguese adults [32] which may reflect a lower intake of water-rich foods and food preparations among the obese.

Although no data on the contribution of food and beverages, other than alcoholic beverages, to total water intake were provided by this research, data from a previous published study conducted in a convenience sample of physically active Portuguese elderly have shown that the contribution of water from different dietary sources did not differ between sexes, except for alcoholic beverages, with a significantly higher percentage contribution in men (10.5% vs. 1.7%, *p* < 0.001) [33]. In the present study there was also a significantly higher prevalence of alcoholic beverage consumption among men (50.5% vs. 27.9% among women, *p* > 0.001). The later finding may contribute to explain that the association between urine osmolality and obesity was only observed among men who may not be able to compensate increased water needs as women apparently do. There is a significantly lower fluid intake and a lower compliance with water intake recommendations among Portuguese adult men, particularly those older than 50 years [34]. In line with this finding, we observed higher average urine osmolality in men compared to women, which probably reflects a worse hydration status.

Elderly subjects have lower 24-h urine osmolality, reflecting the impaired concentrating capacity of the kidney with aging. The larger water volume necessary to excrete a given renal solute load [35] reflected in a lower urine osmolality has been indicated as a contributory factor to a low diagnostic accuracy of urinary biomarkers, namely osmolality, to assess hydration status in older people [36].

In the absence of specific cutoff points of optimal osmolality, particularly in elderly, we opted to stratify the representative sample of Portuguese elderly into tertiles of osmolality, by sex, in order to compare the weight status of individuals according to hydration status, reflected by a higher 24-h urine osmolality. If we would have applied the formula proposed by Manz et al. [11] to classify individuals as hypohydrated or at risk of hypohydration, which takes into account the decreasing urine osmolality with aging, the result of the association between hydration status and weight status was not statistically significant. Despite this, we believe that we should not neglect the positive association between the tertiles of urine osmolality and the occurrence of obesity in men, since the tertiles rank the subjects according to the urine concentration, and the participants belonging to the third tertile present a worse hydration status than the remaining sample.

Regarding the different cut-off points proposed in the literature [12,13,37], we do not consider their use in the elderly as adequate since they do not take into account the renal changes underlying the aging process.

A limitation of this study was the single period of 24-h urine collection, which may not represent an individual’s normal behavior. However, this may also be considered a strength of the present study since the 24-h urine collection is considered the gold standard to assess urine concentration [8], and in the few studies addressing the association between weight status and urine osmolality, spot urine samples were used [13,15]. It has been argued that spot urine samples do not represent the 24-h osmolality since they are unable to predict 24-h sodium excretion, which greatly contributes to urine concentration, at an individual level [38,39] and should not be used for hydration assessment [40]. In addition, having taken into account potential confounders such as age, residence (community-dwelling/institutionalized) or taking diuretics was crucial when studying the association between weight status and urine osmolality and it is another important strength of this study. Although the present study was conducted with a nationwide sample of 1500 older adults, the possibility of type II error cannot be ruled out. The sample size in some strata may not have enough power to detect a statistical significance. Also, the recruitment methodology used in the present study did not allow us to calculate the response rate and the reasons for refusals. Thus, the possibility that this sample can be biased towards a more responsive group of older adults cannot be ruled out.

The available resources and the timeline of the Nutrition UP 65 project did not allow the inclusion of the participants’ food intake assessment, and thus no information on the ingestion of food and beverages is available, besides alcoholic beverage consumption.

Although equations for estimating water requirements in clinical settings are frequently dependent on body weight [41], population level recommendations for total water adequate intake do not vary with weight status [42,43]; notwithstanding, it has been stated that water needs depend on metabolic rate, body surface area and body weight [44] which is in line with the results from the present study that suggests that people with obesity may exhibit increased water needs to compensate a higher urine osmolality.

Several studies have shown a positive effect of increasing water intake on weight reduction and maintenance [45], which reinforces the role of hydration in weight management. Even though causality cannot be established, this cross-sectional evaluation highlights the need for implementing studies in order to clarify the association between hydration and obesity in older men.

## 5. Conclusions

Portuguese older men in the highest urine osmolality tertile showed a two-fold higher risk of being obese. These results suggest that the association between hydration and weight status should be further studied and taken into account in research and clinical contexts.

## Figures and Tables

**Table 1:** Socio-demographic, lifestyle, clinical, anthropometric and nutritional characteristics of 755 older Portuguese women, ≥65 years old, participating in a cross-sectional observational study according to osmolality (mOsm/kg) tertiles.

Participants‘ Characteristics	First, <360.1 mOsm/kg (*n* = 251)	Second, 360.1–484.8 mOsm/kg (*n* = 252)	Third, ≥484.8 mOsm/kg (*n* = 252)	*p*
**Age, years, *n* (%)**				
65–79, *n* (%)	183 (72.9)	177 (70.2)	200 (79.4)	0.055
≥80, *n* (%)	68 (27.1)	75 (29.8)	52 (20.6)	
**Regional area, *n* (%)**				0.002
North	77 (30.7)	85 (33.7)	81 (32.1)	
Center	78 (31.1)	62 (24.6)	48 (19.0)	
Lisbon Metropolitan Area	56 (22.3)	67 (26.6)	79 (31.3)	
Alentejo	17 (6.8)	19 (7.5)	35 (13.9)	
Algarve	11 (4.4)	9 (3.6)	2 (0.8)	
Madeira	9 (3.6)	8 (3.2)	2 (0.8)	
Azores	3 (1.2)	2 (0.8)	5 (2.0)	
**Education, years, *n* (%)**				
No formal education	47 (18.7)	46 (18.3)	32 (12.7)	0.725
1–3	56 (22.3)	52 (20.6)	61 (24.2)	
4	115 (45.8)	119 (47.2)	118 (46.8)	
5–11	23 (9.2)	26 (10.3)	30 (11.9)	
≥12	10 (4.0)	9 (3.6)	11 (4.4)	
**Residence, *n* (%)**				
Community-dwelling	234 (93.2)	237 (94.0)	248 (98.4)	0.013
Institutionalized	17 (6.8)	15 (6.0)	4 (1.6)	
**Marital status, *n* (%)**				
Single/divorced/widowed	168 (66.9)	156 (61.9)	146 (57.9)	0.114
Married/common-law marriage	83 (33.1)	96 (38.1)	106 (42.1)	
**Household income, €, *n* (%)**				
<500	61 (24.3)	50 (19.8)	54 (21.4)	0.795
500–999	55 (21.9)	60 (23.8)	55 (21.8)	
≥1000	20 (8.0)	25 (9.9)	29 (11.5)	
Does not know or does not declare	115 (45.8)	117 (46.4)	114 (45.2)	
**Physical activity (IPAQ), kcal/week, *n* (%)**				
Normal	211 (84.1)	198 (78.6)	219 (86.9)	0.039
Low	40 (15.9)	54 (21.4)	33 (13.1)	
**Tobacco use, *n* (%)**				
No	248 (98.8)	249 (98.8)	247 (98.0)	0.693
Yes	3 (1.2)	3 (1.2)	5 (2.0)	
**Alcoholic beverage consumption, number of drinks ^a^**				
None	181 (72.1)	176 (70.1)	187 (74.2)	
Moderate (women = 1/day; men = 1 or 2/day)	48 (19.1)	55 (21.9)	47 (18.7)	0.828
Heavy (women ≥ 2/day, men ≥ 3/day)	22 (8.8)	20 (8.0)	18 (7.1)	
**Cognitive performance (MMSE), *n* (%)**				
Maintenance	238 (94.8)	235 (93.3)	237 (94.0)	0.759
Impairment	13 (5.2)	17 (6.7)	15 (6.0)	
**Number of medicines and supplements, *n* (%)**				
0	43 (17.1)	36 (14.3)	43 (17.1)	0.248
1–4	105 (41.8)	121 (48.0)	127 (50.4)	
≥5	103 (41.0)	95 (37.7)	82 (32.5)	
**Diuretics, *n* (%)**				
No	205 (81.7)	202 (80.2)	215 (85.3)	0.295
Yes	46 (18.3)	50 (19.8)	37 (14.7)	
**BMI, kg/m^2^, *n* (%)**				
Underweight/normal	36 (14.3)	37 (14.7)	29 (11.5)	0.111
Overweight	117 (46.6)	102 (40.5)	95 (37.7)	
Obesity	98 (39.0)	113 (44.8)	128 (50.8)	
**Nutritional status (MNA^®^-SF), *n* (%)**				
Not undernourished	208 (82.9)	206 (81.7)	211 (83.7)	0.839
Undernutrition risk/undernourished	43 (17.1)	46 (18.3)	41 (16.3)	
**Season urine sample collection, *n* (%)**				
Autumn/Winter	162 (64.5)	164 (65.1)	151 (59.9)	0.419
Spring/Summer	89 (35.5)	88 (34.9)	101 (40.1)	

IPAQ: International Physical Activity Questionnaire; MMSE: Mini Mental State Examination; MNA^®^-SF: Mini Nutritional Assessment^®^-Short Form. ^a^ One missing value because participant could not recall the frequency of consumption.

**Table 2:** Socio-demographic, lifestyle, clinical, anthropometric and nutritional characteristics of 563 older Portuguese men, ≥65 years old, participating in a cross-sectional observational study according to osmolality (mOsm/kg) tertiles.

Participants‘ Characteristics	First, <433.2 mOsm/kg (*n* = 188)	Second, 433.2–602.1 mOsm/kg (*n* = 188)	Third, ≥602.1 mOsm/kg (*n* = 187)	*p*
**Age, years, *n* (%)**				0.001
65–79, *n* (%)	133 (71.1)	158 (84.0)	161 (85.6)	
≥80, *n* (%)	54 (28.9)	30 (16.0)	27 (14.4)	
**Regional area, *n* (%)**				0.514
North	54 (28.9)	59 (31.4)	68 (36.2)	
Center	48 (25.7)	48 (25.5)	49 (26.1)	
Lisbon Metropolitan Area	57 (30.5)	46 (24.5)	41 (21.8)	
Alentejo	14 (7.5)	20 (10.6)	18 (9.6)	
Algarve	9 (4.8)	9 (4.8)	3 (1.6)	
Madeira	2 (1.1)	4 (2.1)	4 (2.1)	
Azores	3 (1.6)	2 (1.1)	5 (2.7)	
**Education, years, *n* (%)**				
No formal education	18 (9.6)	19 (10.1)	11 (5.9)	0.801
1–3	25 (13.4)	24 (12.8)	25 (13.3)	
4	99 (52.9)	102 (54.3)	114 (60.6)	
5–11	33 (17.6)	32 (17.0)	30 (16.0)	
≥12	12 (6.4)	11 (5.9)	8 (4.3)	
**Residence**				
Community-dwelling	176 (94.1)	186 (98.9)	187 (99.5)	0.001
Institutionalized	11 (5.9)	2 (1.1)	1 (0.5)	
**Marital status ^a^, *n* (%)**				
Single/divorced/widowed	79 (42.2)	69 (36.9)	48 (25.5)	0.002
Married/common-law marriage	108 (57.8)	118 (63.1)	140 (74.5)	
Household income, *n* (%)				
<500	16 (8.6)	19 (10.1)	18 (9.6)	0.308
500–999	44 (23.5)	44 (23.4)	30 (16.0)	
≥1000	25 (13.4)	29 (15.4)	39 (20.7)	
Does not know or does not declare	102 (54.5)	96 (51.1)	101 (53.7)	
**Physical activity ^b^ (IPAQ), kcal/week, *n* (%)**				
Normal	157 (84.4)	158 (84.5)	172 (91.5)	0.067
Low	29 (15.6)	29 (15.5)	16 (8.5)	
**Tobacco use, *n* (%)**				
No	173 (92.5)	171 (91.0)	172 (91.5)	0.858
Yes	14 (7.5)	17 (9.0)	16 (8.5)	
**Alcoholic beverage consumption, number of drinks ^c^**				
None	87 (46.5)	99 (52.7)	92 (49.2)	0.359
Moderate (women = 1/day; men = 1 or 2/day)	77 (41.2)	62 (33.0)	63 (33.7)	
Heavy (women ≥ 2/day, men ≥ 3/day)	23 (12.3)	27 (14.4)	32 (17.1)	
**Cognitive performance (MMSE), *n* (%)**				
Maintenance	177 (94.7)	182 (96.8)	182 (96.8)	
Impairment	10 (5.3)	6 (3.2)	6 (3.2)	0.462
**Number of drugs & supplements in 24-h, *n* (%)**				
0	42 (22.5)	43 (22.9)	40 (21.3)	0.614
1–4	101 (54.0)	105 (55.9)	115 (61.2)	
≥5	44 (23.5)	40 (21.3)	33 (17.6)	
**Diuretics, *n* (%)**				
No	154 (82.4)	166 (88.3)	176 (93.6)	0.003
Yes	33 (17.6)	22 (11.7)	12 (6.4)	
**BMI, kg/m^2^, *n* (%)**				
Underweight/normal	38 (20.3)	32 (17.0)	29 (15.4)	0.144
Overweight	97 (51.9)	101 (53.7)	86 (45.7)	
Obesity	52 (27.8)	55 (29.3)	73 (38.8)	
**Nutritional status (MNA), *n* (%)**				
Not undernourished	162 (86.6)	172 (91.5)	166 (88.3)	0.316
Undernutrition risk/undernourished	25 (13.4)	16 (8.5)	22 (11.7)	
**Season urine sample collection ^d^, *n* (%)**				
Late Autumn/Winter	69 (37.1)	66 (35.3)	54 (28.7)	0.196
Spring/Summer	117 (62.9)	121 (64.7)	134 (71.3)	

IPAQ: International Physical Activity Questionnaire; MMSE: Mini Mental State Examination; MNA^®^-SF: Mini Nutritional Assessment^®^-Short Form. ^a^ One missing value because one participant preferred not to declare his marital status; ^b^ Two missing values because energy spent in physical activities could not be computed due to missing data; ^c^ One missing value because participant could not recall the frequency of consumption; ^d^ Two missing values because the Laboratory was not able to provide this information for two participants.

**Table 3:** Multinomial logistic regression results for the association between tertiles of osmolality (mOsm) and body mass index (kg/m^2^) for 1318 Portuguese older adults, ≥65 years old, participating in a cross-sectional observational study.

Participants’ Characteristics	Second Tertile ^a^		Third Tertile ^b^	
	Crude OR (95% CI)	Adjusted OR (95% CI)	Crude OR (95% CI)	Adjusted OR (95% CI)
**Women ^c^ (*n* = 755)** **BMI (kg/m^2^)**				
Underweight/normal	1	1	1	1
Overweight	0.85 (0.50–1.44)	0.84 (0.49–1.43)	1.01 (0.58–1.76)	0.96 (0.54–1.71)
Obesity	1.12 (0.66–1.91)	1.09 (0.63–1.86)	1.62 (0.93–2.83)	1.52 (0.86–2.70)
**Regional area**				
North	1.66 (0.27–10.2)	1.85 (0.30–11.4)	0.63 (0.15–2.73)	0.78 (0.18–3.43)
Center	1.19 (0.19–7.36)	1.31 (0.21–8.13)	0.37 (0.08–1.62)	0.46 (0.10–2.06)
Lisbon Metropolitan Area	1.80 (0.29–11.1)	1.93 (0.31–12.0)	0.85 (0.19–3.69)	1.04 (0.24–4.62)
Alentejo	1.68 (0.25–11.3)	1.89 (0.28–12.8)	1.24 (0.26–5.79)	1.40 (0.29–6.66)
Algarve	1.23 (0.17–9.02)	1.29 (0.18–9.55)	0.11 (0.01–0.87)	0.13 (0.02–1.02)
Madeira	1.33 (0.18–10.1)	1.58 (0.18–9.55)	0.13 (0.02–1.09)	0.15 (0.02–1.28)
Azores	1	1	1	1
**Residence type**				
Community-dwelling	1.15 (0.56–2.35)	1.20 (0.57–2.49)	4.50 (1.49–13.6)	3.99 (1.30–12.2)
Institutionalized	1	1	1	1
**Physical activity (kcal)**				
Normal	0.70 (0.44–1.09)	0.70 (0.44–1.11)	1.26 (0.76–2.07)	1.25 (0.75–2.10)
Low	1	1	1	1
**Men ^d^ (*n* = 563)** **BMI**				
Underweight/normal	1	1	1	1
Overweight	1.24 (0.72–2.14)	1.24 (0.71–2.18)	1.16 (0.66–2.04)	1.18 (0.66–2.11)
Obesity	1.26 (0.69–2.38)	1.31 (0.71–2.43)	1.84 (1.01–3.35)	1.97 (1.06–3.66)
**Age (years)**				
65–79	2.14 (1.29–3.53)	1.81 (1.07–3.06)	2.42 (1.45–4.06)	1.71 (0.99–2.96)
≥80	1	1	1	1
**Residence Type**				
Community-dwelling	5.81 (1.27–26.6)	4.65 (1.00–21.7)	11.7 (1.49–91.5)	8.07 (1.00–64.9)
Institutionalized	1	1	1	1
**Marital Status**				
Single/divorced/widowed	0.80 (0.53–1.21)	0.87 (0.57–1.34)	0.47 (0.30–0.73)	0.50 (0.32–0.79)
Married/common-law marriage	1	1	1	1
**Diuretics**				
No or unknown use	1.62 (0.90–2.90)	1.44 (0.78–2.65)	3.14 (1.57–6.30)	2.99 (1.45–6.17)
Use	1	1	1	1

^a^ 360.1–484.8 mOsm/kg for women (*n* = 252); 433.2–602.1 mOsm/kg for men (*n* = 188); ^b^ ≥484.8 mOsm/kg for women (*n* = 251); ≥602.1 mOsm/kg for men (*n* = 187); ^c^ Variables: regional area (categories North; Center; Lisbon Metropolitan Area; Alentejo; Algarve; Madeira; reference: Azores); residence type (categories community-dwelling; reference: institutionalized); physical activity (categories normal, ≥270 kcal/week; reference: low, <270 kcal/week); ^d^ Variables: age (categories 65–79 years; reference: ≥80 years); residence type (categories community-dwelling; reference: institutionalized); marital status (categories single, divorced, widowed; reference: married or common-law marriage); diuretics (no or unknown use; reference: use).
